# Elevated Intraocular Pressure due to Arteriovenous Fistula between External Carotid Artery and Facial Vein

**DOI:** 10.1155/2014/897928

**Published:** 2014-10-02

**Authors:** Halil Huseyin Cagatay, Metin Ekinci, Selam Yekta Sendul, Ceylan Uslu, Mehmet Demir, Sıtkı Mert Ulusay, Ender Uysal, Selma Şeker

**Affiliations:** ^1^Department of Ophthalmology, Faculty of Medicine, Kafkas University, Merkez, 36100 Kars, Turkey; ^2^Department of Ophthalmology, Şişli Etfal Training and Research Hospital, 34371 Istanbul, Turkey; ^3^Department of Ophthalmology, Çerkezköy State Hospital, 59500 Tekirdağ, Turkey; ^4^Department of Radiology, Kars State Hospital, 36000 Kars, Turkey; ^5^Department of Radiology, Şişli Etfal Training and Research Hospital, 34371 Istanbul, Turkey; ^6^Department of Otorhinolaryngology, Terme State Hospital, 55600 Samsun, Turkey

## Abstract

Aqueous outflow via the conventional outflow pathway is dependent on the pressure gradient between intraocular pressure (IOP) and episcleral venous pressure (EVP). Elevated IOP resulting from increased EVP is a well-known complication of arteriovenous fistulas, which are usually between the carotid artery and the cavernous sinus. Arteriovenous malformations usually occur spontaneously, after a trauma or from iatrogenic causes, and they manifest with findings of chemosis, dilatation of the conjunctival vessels, exophthalmos, and extraocular motility limitation. In this study, we present a case of elevated IOP due to facial arteriovenous malformations following a functional neck dissection surgery that caused intraocular pressure elevation.

## 1. Introduction

Acquired arteriovenous fistulas (AVFs) are mostly caused by trauma or surgery; these lesions can involve all parts of the body [1–5]. AVF results in an increase in venous return, pressure, and volume. The signs and symptoms of a large AVF include pulsatile swelling, systolic murmur, palpable thrill, and dilated superficial veins. Cases with secondary glaucoma or elevated intraocular pressure that occurred as a result of a carotid or cavernous fistula have been previously reported [1–6]. To our knowledge, this is the first reported case of a secondary open angle glaucoma due to carotid-facial vein fistula as a complication of functional neck dissection surgery. Written informed consent was obtained from the patient.

## 2. Case Presentation

**Figure 1:**
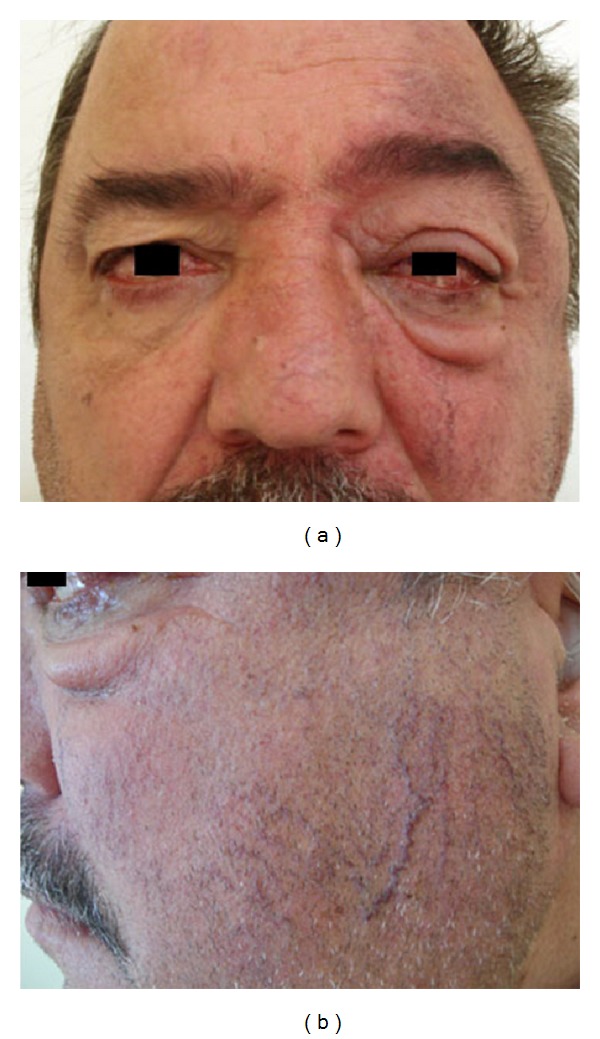
Patient with conjunctival chemosis, edema of the eyelid, dilatation of the conjunctival and episcleral vessels, and more visible superficial skin vessels on the left side of the face.

**Figure 2:**
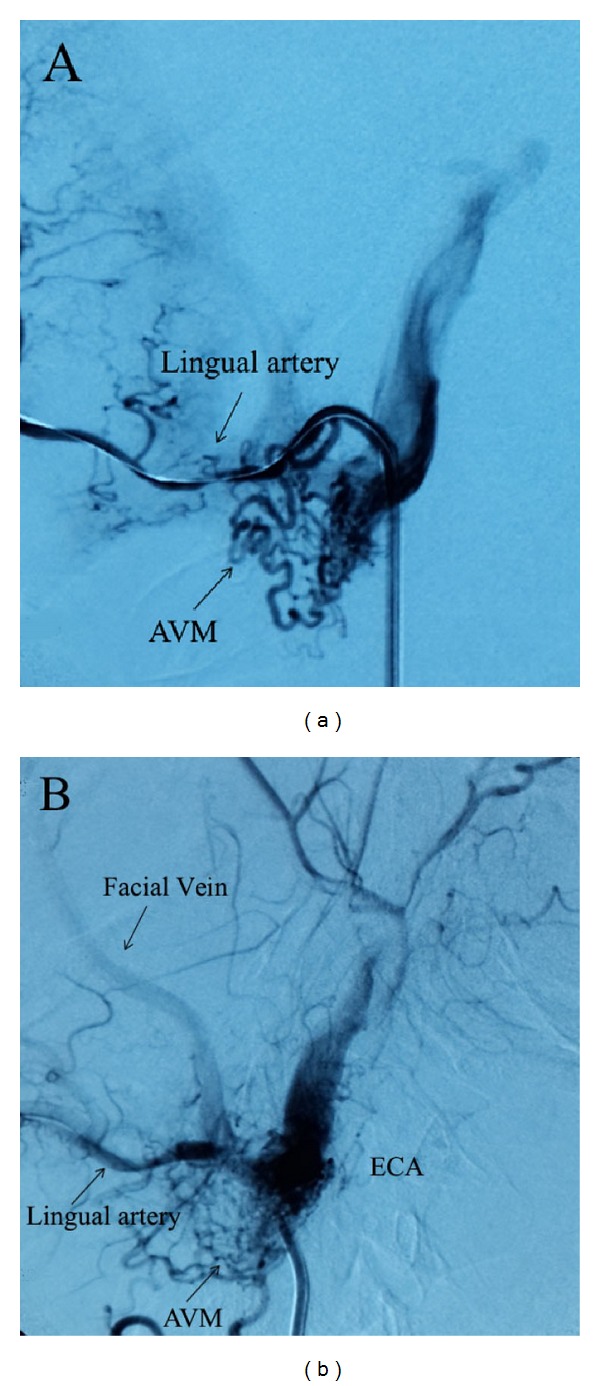
(a) Left external carotid artery and selective lingual artery catheterisation; digital subtraction angiography showed multiple fistulas originating from the lingual artery and draining into the facial vein. (b) Facial vein showed a filling towards the cranium. ECA: external carotid artery; AVM: arteriovenous malformation.

**Figure 3:**
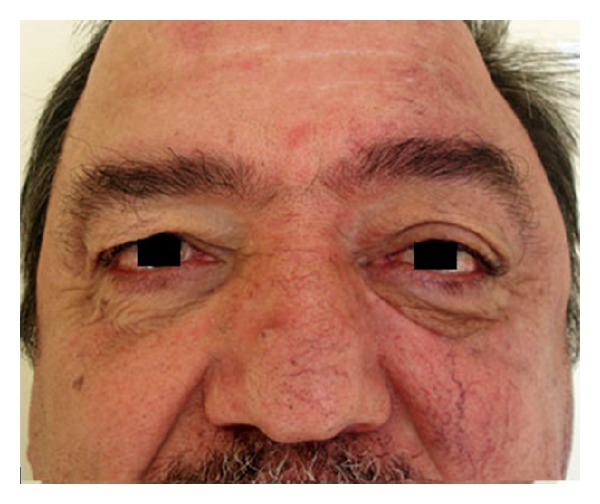
Recovery of the patient after the ligation of fistulas via open approach.

**Figure 4:**
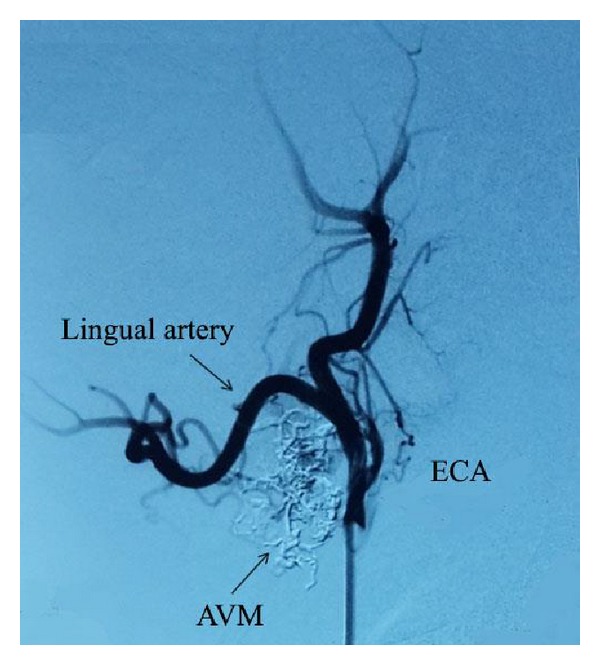
After the treatment digital subtraction angiography demonstrated that the arteriovenous malformations were completely occluded.

A 49-year-old male applied to our clinic with complaints of epiphora and swelling in the left eye that had begun approximately 3 years ago. A complete ophthalmic examination was done and the best corrected visual acuity was 20/20 in both eyes. Intraocular pressure was 14 mmHg in the right eye and 33 mmHg in the left eye. The patient had conjunctival chemosis, edema of the eyelid, and dilatation of the conjunctival and episcleral vessels. In addition, the superficial skin vessels on the left side of the face were more visible (Figure 1). The anterior segment and fundus were unremarkable, except for a hyperemic disc. Gonioscopy revealed an open angle in both eyes. Automated perimetry (30/2) showed no visual field defects. A retinal nerve fiber analyzer revealed normal neuroretinal rim thickness in both eyes. Pachymetry showed a central corneal thickness of 556 nm OD and 560 nm OS. The patient had undergone neck surgery five years ago and did not have a history of radiation therapy. Topical antiglaucomatous medication including a prostaglandin analogue and a combined preparation of dorzolamide and timolol maleate were administered to the left eye and three weeks later the IOP of the left eye was 28 mmHg. In digital subtraction angiography (DSA) imaging, multiple fistulas were detected between the sublingual artery and the facial vein (Figure 2). When the intravascular approach was unsuccessful in treating the multiple fistulas, surgical intervention was performed and the fistulas were ligated via an open approach. At the second month of follow-up, eyelid edema, chemosis, and IOP elevation had regressed (Figure 3). Complete occlusion of the fistulas was demonstrated on follow-up angiography (Figure 4). Continued monitoring of the visual fields and the retinal nerve fiber layer taken by optical coherence tomography (OCT) showed general stability in both eyes through the 3-year follow-up period.

## 3. Discussion

Intraocular pressure is influenced by the production and outflow of aqueous humor [7–9]. Outflow is dependent on the difference between the intraocular pressure (IOP) and episcleral venous pressure (EVP). Aqueous humor exits the eye through the trabecular meshwork into Schlemm's canal and flows through 25–30 collector canals into the episcleral veins, superior inferior ophthalmic veins, cavernous sinus, petrosal sinuses, and internal and external jugular veins. Uveoscleral flow should be calculated using the Goldman equation [10]. Episcleral venous pressure elevation may occur due to arteriovenous malformations (AVM), venous obstructions, or idiopathically [3, 4, 6–10]. Most venous drainage of the orbit occurs posteriorly to the cavernous sinus, and usually cavernous sinus-related vascular pathologies can cause an elevation of the EVP and IOP. Previously, it has been shown that anterior orbit and facial vascular pathologies may also cause an elevation of the EVP and IOP [11]. Facial and episcleral hemangiomas in Sturge Weber syndrome are well known vascular abnormalities which may cause increased EVP [12].

The clinical presentation of our case was similar to indirect CCFs; yet the absence of proptosis and presence of hyperpigmentation on the left side of the face were different from indirect CCFs. When we considered the medical history of the patient and the signs of venous abnormalities, we performed magnetic resonance angiography and DSA. Angiography showed arteriovenous malformations between the lingual artery and the facial vein and retrograde flow from the facial vein to the angular vein that caused an elevation of intraocular pressure. After failure to treat the multiple fistulas via the endovascular approach, the fistulas were ligated via the open approach. Surgical intervention was carried out and the patient recovered uneventfully in 10 days.

Although endovascular (EVT) techniques are the first choice of treatment, they may occasionally fail, as in the present case. In our case, when DSA was performed, it revealed that the complexity of the multiple fistulas would not allow successful treatment using EVT techniques.

In conclusion, this case shows that facial AVFs may mimic indirect CCFs and cause an increase in intraocular pressure; it is possible to decrease IOP completely by treating the AVFs.
